# On the Diagnostic Value of Expectoration and Pain in Pulmonary Affections

**Published:** 1848-07

**Authors:** 


*On the Diagnostic value of Expectoration and Pain in Pulmonary affections.* By Prof. ANDRAL.

Let us now enquire into the diagnostic value of expectoration in various maladies.

In acute laryngitis, simple or puriform mucus may be rejected: when a certain quantity of pus is suddenly thrown up, it points to a submucous abscess of the larynx; false membranes secreted in the larynx may also be seen, as in variola, for instance. In chronic laryngitis, blood, expectorated in small quantities, indicates an ulcer of the mucous membrane; if the amount of blood be at all considerable, its source is not in the larynx. A chronic form of croup has been described in which the patients cough up false membranes, and this circumstance often exercises a most favorable influence upon the progress of the malady. Polypous concretions, and vegetations formed in the laryngeal cavity, have been rejected; also pus from an abscess developed in the organ, as in a case published by Dr. Pravaz, of Lille: a tumour, soft and fluctuating, had been previously recognized by the introduction of the finger, and disappeared after the sudden expectoration of a certain quantity of purulent matter, with which two small concretions were also thrown up. The same author relates in his inaugural thesis a case in which two hydatids were found in the ventricles of the larynx, but we have never ourselves observed any instance of the kind.

Acute bronchitis is not in its first period attended with expectoration; but, as the disease progresses, the cough becomes loose; and a colorless, transparent mucus, more or less viscid in its nature, is rejected, occasionally streaked with blood if the cough be violent. This matter gradually becomes less transparent, and acquires a puriform appearance, decreasing at the same time in abundance, in proportion as the case progresses towards a favorable termination. In some patients the expectoration is puriform from the first, in others it never assumes that character. Occasionally, even the cough is dry from the beginning to the end of the disease. In chronic bronchitis, the expectoration usually consists of a slimy fluid, more or less puriform in appearance: when dyspnœa is present, this matter is very viscid, and generally spumous. It is sometimes constituted by a homogeneous liquid, which bears the greatest resemblence to the expectoration in consumption, from which neither chemistry nor the microscope can distinguish it. The abundance of the rejected fluids may be very considerable, particularly in the aged, the general condition of the system suffering at the same time much less than it would, *a priori*, seem rational to suppose. The odor of the matter is not characteristic, and may accidently acquire great fetidity. In some cases of chronic inflammation of the bronchi, no expectoration is observed; in others a peculiarly granular mucus is thrown up, which has a remarkable tendency to solidity; and in both these cases, pulmonary emphysema often follows bronchitis.

During the first two days of pneumonia, characteristic expectoration is seldom noticed, but after this period the rejected matter is viscid, transparent, spumous, and rusty in color; as the disease advances, the fluid becomes darker and less frothy, and gradually resumes its natural hue if the malady terminates favorably; if, on the contrary, it becomes darker and more viscid, or brown, and more fluid, the prognosis acquires greater gravity. It has been often said that where the colour of the expectoration resembled that of the juice of stewed prunes, the circumstance indicated diffused suppuration of the lung; we believe that it points more to increased severity of the case than to that particular fact. In the aged, the expectoration is often of a reddish grey, and may completely be suspended some time before a fatal issue, either from a real cessation of the morbid secretion, or more frequently from loss of power to reject it. In the pneumonia of children no expectoration takes place, and when this disease is a complication of other ailments, consumption for instance, the rejected matter is often deprived of its characteristic appearances. M. Remack, of Berlin, states ("Archives," January, 1846), that in the expectoration of pneumonia, small concretions are rejected, constituted by fibrine, and incarcerating purulent corpuscles in their network; the shape of these concretions is the same as that of the bronchi: we have several times assured ourselves of the accuracy of this observation, which may assist considerably the diagnosis of doubtful cases. Chronic pneumonia presents no special expectoration.

Œdema pulmonis and emphysema are not attended with any characteristic appearances in this respect. At the beginning of attacks of asthma, the expectoration is usually suppressed, and returns at the close of the paroxysms. Pulmonary appoplexy usually causes hemorrhage and the rejection of a small quantity of blood, but not constantly. In gangrene of the lung, the sputa are thin, dark, and extremely fetid.

Pulmonary consumption at its various periods occasions an expectoration, the characters of which it is important to be well acquainted with. Mucus, pus, blood, concretions, &c., may be rejected, and their relative signification should be clearly understood. During the first period of phthisis, the cough is frequently dry, or followed merely by the expulsion of pure mucus; blood is often rejected at this period, with the characters which we have elsewhere described. Calculi, also, may be expectorated, formed of crude tubercular matter. When softening occurs, the appearances are usually those observed in bronchitis, the sputa being sometimes streaked with a substance of a yellowish, dead-white color, supposed to consist of detached tubercular secretion: also, occasionally granular agglomerations of tubercle are found floating in the mucus; hæmoptysis may occur at this stage; but when cavities have formed in the lung the appearances are more characteristic. A large tubercular mass may be expelled suddenly, but in general its elimination is gradual. The discharge is thus formed of three parts, viz: bronchial mucus, tubercular matter, and a purulent fluid secreted by the walls of the accidental cavity. According to the relative proportions of these three elements the expectoration varies in its characters. In most subjects it separates into two layers—a semi-transparent, gummy fluid, and a more opaque, solid matter. The latter may be suspended in flakes, or float on the surface of the fluid in small round patches (nummular expectoration.) At a still later period of the disease, the semi-transparent matter is no more to be found, and the matter consists merely of a thick, purulent fluid. At this stage of phthisis, hæmoptysis is more rare than in the first periods. The odor or taste of the expectoration are not characteristic, in spite of the Hippocratic aphorism, that in consumption it is at first salt, and afterwards sweetish. The quantity rejected varies greatly, not only in several, bnt even in the same individual. Shortly before a fatal termination the expectoration may be suppressed, or become looser from the enlargement of the caverns. Microscopic examination shows in the sputa of consumption the presence of pus and blood corpuscles, of epithelium, of false membranes, and of pulmonary tissue. When a piece of tubercular lung is examined with the microscope numerous corpuscles can be seen, with the particular form described in the first series of these lectures. But it is very uncommon to meet with these special granulations in the sputa, being, as it would seem, dissociated before they are expelled from the chest by the efforts of coughing. It has been said, and it is quite true, that tnbercular sputa sink in water, and burn with a flame when desiccated; but these characters cannot distinguish them from the sputa of bronchitis, because they result from the presence of pus, which may exist in one disease as well as in the other.

When hydatids form in the pulmonary organs, they may be expelled by expectoration. The sputa of cancer of the lung are said to resemble gooseberry jam in color and consistency, but this fact requires further confirmation.

In diseases of the pleura, the expectoration is usually absent, or resembles that of bronchitis; but pleuritic effusions may suddenly be discharged by the bronchi, after ulceration of the surface of the lung. True pus is thus rejected, and penetration of air into the pleuritic cavity soon communicates to its contents a fetid alliaceous odor. The consecutive discharge may continue for a considerable time, and may be recalled after temporary cessation by change of position of the subject, by which a change is also effected in the relative situations of the pleuritic effusion and of the pulmonary fistula. Even after long duration, these cases are susceptible of recovery.

In diseases of the pulmonary organs, the sensibility of the patient may be modified, and furnish signs for the diagnosis.

Many acute or chronic diseases of the larynx may exist without pain, and present a striking contrast between the great change of the voice and the total absence of suffering. When pain accompanies ulcers of the larynx, it is more marked when the sore occupies the vocal chords, or is situated above them, than when it is placed below the ventricles. Affections of the trachea are seldom painful. Acute bronchitis is usually attended with a distressing sense of heat within the chest, which increases with the cough, or with the inspiration of cold air. This pain may occupy the sternal region, or be complained of between the shoulders. Pneumonia like other inflammations of parenchymatous organs, is seldom of itself a painful disease. "Pulmonum inflammatio," says Boerhaave, "plus affert periculi quam doloris;" a perfectly accurate remark, the stitch in the side, which so usually accompanies pneumonia, being in most cases due to pleuritic complication. Many consumptive patients suffer very little, others, on the contrary, are frequently troubled with pain, due to the participation of the pleura in the disorder—a fact demonstrated by the adhesions found within the thorax of these subjects on *post-mortem* examination. Some patients feel a sort of tightness round the chest, and are fully aware, from the nature of the sensation, that their respiration is not complete; in others a sense of uneasiness is complained of in the superior part of the chest; and in these cases, on dissection, caverns are often found in these regions, separated from the pleura by very thin walls. Occasionally percussion of the chest, and even auscultation with the stethoscope, cause pain in phthisis. Pain between the shoulders is very generally looked upon as a sign of consumption; we believe that the importance of this symptom has been much overrated; it seems to us to be merely a muscular pain, due to debility, and observable in most cases of chronic disease; it is, for instance, almost constant in chlorosis. Hydatids and cancer of the respiratory organs are productive of suffering only when the pleura is affected. Acute pleuritis seldom exists without pain; its intensity is variable from a trifling to a most intense stitch in the side. It is usually seated below the breast on the affected side, and increased by motion, pressure, cough, or inspiration. It may extend to the whole thoracic wall, or be limited to the cartilaginous edges of the ribs; in diaphragmatic pleurisy, it spreads to the epigastrium, and even to the abdominal parietes. An ingenious explanation of this pain has been offered by Dr. Beau, and seems confirmed by anatomical investigation. That gentleman attributes it to inflammation of the intercostal nerves, which, in the posterior third of their course, lie in close apposition with the pleura. Irritation of a nerve, causing pain to be felt in the region of its terminal distribution, excitement of the posterior part of these nerves in pleurisy, naturally occasions a suffering which is referred to the anterior region of the thorax, immediately below the breast.—*Jour. Med. Times. Reported by Dr. McCarthy, Paris.*

